# Collaborative roles of Temporoparietal Junction and Dorsolateral Prefrontal Cortex in Different Types of Behavioural Flexibility

**DOI:** 10.1038/s41598-017-06662-6

**Published:** 2017-07-25

**Authors:** Shisei Tei, Junya Fujino, Ryosaku Kawada, Kathryn F. Jankowski, Jukka-Pekka Kauppi, Wouter van den Bos, Nobuhito Abe, Genichi Sugihara, Jun Miyata, Toshiya Murai, Hidehiko Takahashi

**Affiliations:** 1 Department of Psychiatry, Kyoto University Graduate School of Medicine, Sakyo-ku, Kyoto 606-8507 Japan; 2 Institute of Applied Brain Sciences, Waseda University, 2-579-15 Mikajima, Tokorozawa, Saitama 359-1192 Japan; 3 School of Human and Social Sciences, Tokyo International University, Saitama, 350-1198 Japan; 4 Medical Institute of Developmental Disabilities Research, Showa University Karasuyama Hospital, Tokyo, 157-8577 Japan; 5 Department of Psychology, University of Oregon, Eugene, OR 97403 USA; 6 Department of Mathematical Information Technology, University of Jyväskylä, Jyväskylä, Finland; 7 Department of Computer Science and HIIT, University of Helsinki, P.O. 68 (Gustaf Hällströmin katu 2b), FI-00014 Helsinki, Finland; 8 Center for Adaptive Rationality, Max Planck Institute for Human Development, 14197 Berlin, Germany; 9 Kokoro Research Center, Kyoto University, Sakyo-ku, Kyoto 606-8507 Japan

## Abstract

Behavioural flexibility is essential for everyday life. This involves shifting attention between different perspectives. Previous studies suggest that flexibility is mainly subserved by the dorsolateral prefrontal cortex (DLPFC). However, although rarely emphasized, the temporoparietal junction (TPJ) is frequently recruited during flexible behaviour. A crucial question is whether TPJ plays a role in different types of flexibility, compared to its limited role in perceptual flexibility. We hypothesized that TPJ activity during diverse flexibility tasks plays a common role in stimulus-driven attention-shifting, thereby contributing to different types of flexibility, and thus the collaboration between DLPFC and TPJ might serve as a more appropriate mechanism than DLPFC alone. We used fMRI to measure DLPFC/TPJ activity recruited during moral flexibility, and examined its effect on other domains of flexibility (economic/perceptual). Here, we show the additional, yet crucial role of TPJ: a combined DLPFC/TPJ activity predicted flexibility, regardless of domain. Different types of flexibility might rely on more basic attention-shifting, which highlights the behavioural significance of alternatives.

## Introduction

Behavioural flexibility is the ability to adjust one’s goals to face new situations^1^. This involves shifting attention between different perspectives or decision-rules, and thinking about these conflicting perspectives simultaneously^2^. Attention-shifting thus enables viewing incompatible perspectives, thereby adjusting behavioural goals. Moreover, evidence has suggested that attention-shifting is not only involved in rapidly adjusting responses, but also in selectively maintaining similar responses, because novel changes in a situation can be either favourable or unfavourable to the outcomes of one’s decisions^3^. Therefore, flexibility may best represent a balance between maintaining and shifting between decision rules^4^.

Previous research suggests that behavioural/cognitive flexibility is subserved by a common prefrontal cognitive control system. Specifically, the dorsolateral prefrontal cortex (DLPFC) supports maintaining current decision rules to regulate on-line processing, i.e., sustaining goal-directed attention to filter out irrelevant stimuli and divert attention from incongruent information^5^. However, at the same time, the right temporoparietal junction (R-TPJ) may support flexibility by shifting between decision rules (shapes and colours) at the perceptual level^6^. In fact, although rarely emphasized, TPJ is frequently observed during flexible behaviour, together with DLPFC^7–9^. However, the exact role of TPJ, as well as the collaboration between DLPFC and TPJ, have not been sufficiently explored.

The question arises: how does the link between DLPFC-TPJ support flexible behaviours? In other words, is there a common communication that assists diverse types of flexibility? While it has been proposed that switching function supports perceptual/visual flexibility by co-activating frontal and parietal regions^10^, it is unknown whether this switching applies to higher-order flexibility (e.g., economic and moral flexibility requiring rule switching). Higher-order flexibility has been argued to crucially rely on the cognitive system within PFC that subserves broad set-shifting of perspectives/decision-strategies, and strong top-down biasing for conflict reduction^1, 11^. Incidentally, while TPJ’s subdivisions may subserve distinct functions such as attention-shifting and mentalizing^12–14^, recent meta-analyses have argued for overlapping functions within TPJ, and have speculated about a more general function in social processing^15, 16^. In this respect, we were interested in the shared role of TPJ that may provide a more plausible mechanistic explanation. We hypothesized that TPJ activity during diverse flexibility tasks plays a common role in stimulus driven attention-shifting, thereby contributing to different types of flexibility, and thus the collaboration between DLPFC and TPJ might serve as a more appropriate mechanism than DLPFC alone.

We studied the mediating effect of R-TPJ activity on the relationship between R-DLPFC activity and flexibility across three distinct domains (moral, economic, and perceptual), which are all involved in thinking about conflicting perspectives simultaneously^2^. We measured flexibility when subjects adopted alternative perspectives (i.e., profit/welfare maximization for moral and economic flexibility via considering their conflicting dilemmas). That is to say, moral flexibility was measured by change rates (CR) during a moral-dilemma (MD) task. One CR meant that subjects chose ‘wrong’ when they evaluated whether moral actions were right or wrong (R/W), but flexibly changed to ‘yes’ when they evaluated whether they would or would not conduct the same actions via cost-benefit (C/B) considerations through attention-shifting^17^. Thus, R/W represented subjects’ simple evaluations of whether situations were morally right or wrong, while C/B represented evaluations of how subjects would act in the real world by thinking about these perspectives simultaneously (R/W and C/B), which required greater flexibility. More specifically, C/B primarily induced the moral dilemma: 1) right or wrong perspectives to follow the social norm (rule-based); and 2) cost or benefit perspectives to focus on the consequence of moral violation (result-oriented)^17^. As a result, thinking of conducting such moral violation involved flexible switching of these conflicting perspectives simultaneously, prompting potential shifting to alternative decision choice for welfare maximization^17^. Indeed, flexible or less rigid people are more likely to take result-oriented options^18^. Moral scenarios were designed so that subjects would feel that the scenarios were morally ‘wrong’, but that it was still potentially acceptable to conduct these actions. We thus manipulated the behavioural significance of alternatives by changing the scenarios’ context. The economic flexibility was measured by the acceptance rates (AR) of unfair offers during the Ultimatum Game (UG). Similar to MD, UG induced an economic dilemma: 1) fairness-oriented perspective (to focus on norm violations); and 2) result-based perspective (to focus on monetary reward)^19^. In effect, accepting unfair offers involved flexible switching of these conflicting perspectives simultaneously, which allows shifting towards alternative decisional options. Accordingly, people with more flexibility (or less rigidity), tend to behave capably and opportunistically in UG, being good at playing this game while pursuing self-interest^20, 21^. Faced with unfairness, subjects were inclined to reject unfair offers, but sometimes they strategically and flexibly accepted them in consideration of maximizing financial gains, which is the ultimate goal of the game^21^. Furthermore, perceptual flexibility was measured by the number of categories achieved (CA) during the Wisconsin Card Sorting Test (WCST), which is a well-established task for measuring behavioural flexibility in terms of attention set-shifting^9^.

## Results

### Relationship between the level of flexible behaviour and the strength of brain activity

Behavioural results showed that mean CR, AR, and CA among subjects were: 27.3 ± 15.7%, 41.3 ± 39.9%, and 4.6 ± 1.5, respectively. To study brain activity supporting behavioural flexibility, we examined the conflict resolution contrast (C/B > R/W). In whole-brain analysis, this contrast revealed activity in R-DLPFC and R-TPJ (*p* < 0.01, cluster-level family-wise error (*FWE*) corrected; Supplementary Table S1). Besides the above predefined ROIs, significant brain activities were also observed in the region, including primary visual area, cingulate cortex, frontal and temporal lobe, and cerebellum, at a corrected level for multiple comparisons across the entire brain. However, because these results are beyond the scope of our current study, further analyses are focused on our a priori ROIs. Meanwhile, no significant brain activity was detected in the reverse contrast (R/W > C/B). In the subsequent ROI analysis, we also observed activity in R-DLPFC and R-TPJ (Supplementary Table S2). Using the parameter estimates of these ROIs, R-DLPFC activity correlated positively with R-TPJ activity (*r* = 0.42, *p* = 0.019), and also correlated positively with: 1) CR (*r* = 0.40, *p* = 0.026); 2) AR (*r* = 0.49, *p* = 0.008); and 3) CA (*r* = 0.37, *p* = 0.039). Likewise, R-TPJ activity correlated positively with: 1) CR (*r* = 0.38, *p* = 0.033); 2) AR (*r* = 0.49, *p* = 0.007); and 3) CA (*r* = 0.50, *p* = 0.008).

### Mediation analysis

**Figure 1:**
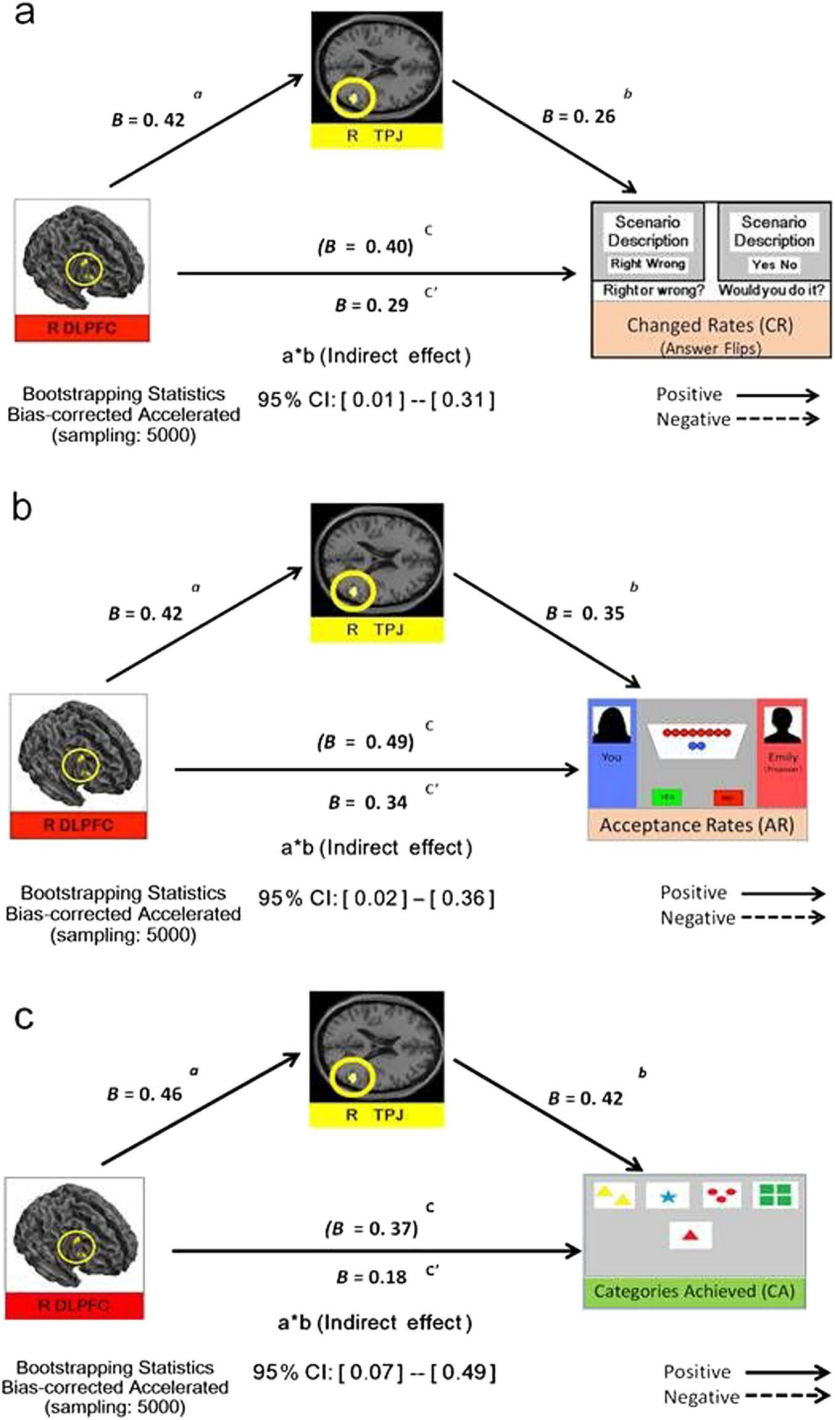
Mediation model for (**a**) moral, (**b**) economic, and (**c**) perceptual domains. R-TPJ was an effective mediator across all domains; there were significant indirect effects on the relationship between R-DLPFC and behavioural flexibility. ‘*B*’ denotes regression coefficients, indicating each link in the model. Confidence intervals (CIs) not including zero indicate indirect effects (*p* < 0.05).

Subsequently, we constructed three mediation models, given the documented role of DLPFC in attention maintenance^5, 22^ and the proposed role of TPJ in collaborating with DLPFC to support attention-shifting^10, 23^. The results showed that R-TPJ activity was a statistically effective mediator of the relationship between R-DLPFC activity and flexibility across all three domains (moral, economic, perceptual; Fig. 1a–c). There were significant indirect effects on the relationship between R-DLPFC and behavioural flexibility (bias-corrected/accelerated, bootstrap 5000; 95% confidence intervals: 0.01–0.31, 0.02–0.36, and 0.07–0.49, respectively). Finally, by averaging the normalized scores of flexibility measures across the three domains (i.e., composited score), there was a statistically significant indirect effect of R-TPJ on the relationship between R-DLPFC and this composited score (bias-corrected/accelerated, bootstrap 5000; 95% confidence intervals: 0.10–0.51).

As an additional analysis, we examined the functional connectivity between R-TPJ and R-DLPFC using psychophysiological interaction analysis (PPI). The results showed that there was a link between the strength of R-TPJ activity and R-DLPFC activity in the conflict resolution contrast [(i.e., C/B > R/W; *p* < 0.01, cluster-level corrected (Monte Carlo simulations^24^); Supplementary Table S3]. Details are described in the Supplementary Results.

## Discussion

We found that TPJ activity not only mediates the relationship between DLPFC activity and moral flexibility during the MD task, but also the relationship between DLPFC activity and other domains of flexibility outside the MRI scanner. In consideration of the established function of TPJ, a key mechanism underlying the link between TPJ activity during a specific task (moral reasoning) that requires higher-order flexibility and lower-order flexibility (perceptual) might be due to basic attention-shifting^14, 25, 26^. Further, TPJ mediated DLPFC and the composited score of flexibility (moral, economic, and perceptual). The collaboration between DLPFC and TPJ might extend across two different types of higher-order flexibility (moral and economic), and this would also support previous findings for its role in lower-order flexibility^10^. One possible interpretation is that such switching represents adjusted attention-shifting: one partly shifts/disengages attention from current decision-rules and explores the significance of alternative choices^27^, thereby updating decision-rules/strategies. DLPFC might send endogenous signals^28^ to modulate TPJ during adaptive disengagement from a current attention-set, which in turn enables TPJ to explore/highlight alternative choices to optimize decision-rules^29, 30^.

The brain activity observed in the conflict resolution contrast supported previous studies regarding flexible behaviours. Our current study observed activity within: prefrontal cortex, posterior cingulate, parietal lobe, in addition to our ROIs (DLPFC and TPJ). Activity including posterior cingulate, superior/middle frontal gyrus, and inferior parietal lobule^7^ had been reported during result-focused flexible decision during MD (cognitive conflict and control)^7^. In addition, according to a review on MD, bilateral DLPFC (Brodmann area/BA 46) and bilateral TPJ/STS (BA 39/22) activities were observed during moral resolution contrast^31^; and distinctive R-TPJ activity (BA 39/40) was also reported^7, 32^. In UG, such value-based decision^20^ was related to DLPFC^8^ and cingulate cortex^33^, which may regulate the emotional response in favour of more flexible decision-making^34^. More specifically, activity including DLPFC (BA 46/9) and bilateral posterior TPJ (BA 39)^35^ were reported in the unfairness > fairness contrast in UG^19^. Likewise, attention set-shifting tasks^36, 37^ including WCST involved executive functioning subserved by DLPFC, cingulate cortex, and superior/inferior parietal lobule^9^, which may support conflict-induced behavioural adjustment^38^. Further, these activities in the prefrontal and parietal regions also support the theory concerning the ventral attention reorienting network^13, 23, 39, 40^. In this line, previous studies on WCST in particular, reported activities including mid-DLPFC (BA 46/9) and anterior-TPJ (BA40)^9, 36, 41^.

The role of attention-shifting in flexibility might apply to the spatial/perceptual domain, as well as to other domains. Indeed, an imbalance between maintaining and shifting between decision rules may induce different types of cognitive inflexibility, such as perseveration (maladaptive focusing on or revisiting particular information) and spatial neglect, of which R-TPJ is the most common substrate^42, 43^. Patients with spatial-neglect are capable of seeing all items in a scene, but have difficulty appropriately allocating attention to these items. In addition, these patients have attentional impairments in non-spatial domains^43^. Neglect syndrome has been ascribed to difficulties in attention allocation in broader domains, such as shifting attention from an external information source to one’s internal on-line presentation or detecting behavioural-significance^42^. In this respect, cognitive ‘inflexibility’ can be regarded as a phenomenon akin to spatial-neglect, whereby one might ignore alternative perspectives/choices due to maladaptive attention allocation, in a manner of cognitive-neglect or mind-blindness^44^. Mind-blindness is a hallmark of autism spectrum disorder (ASD), which is also characterized by atypical R-TPJ functioning and frontoparietal integration^43, 45^. Intriguingly, ASD also shows a broad range of behavioural/cognitive inflexibility, and mind-blindness is linked to impaired spontaneous detection of behavioural-significance^46^.

As an additional analysis, we examined functional connectivity between R-TPJ and R-DLPFC using PPI analysis. The result was in accordance with the discussion stated above. Namely, there was a link between the strength of R-TPJ activity and R-DLPFC activity in the conflict resolution contrast [(C/B > R/W); see Supplementary Results and discussion]. However, the threshold of this result is relatively liberal for arguing robustly about the link between TPJ and DLPFC. Thus, additional experiments especially focusing on the interaction between TPJ and DLPFC should be conducted.

To confirm our interpretation of the results, future research should also examine TPJ activity recruited across each of the three domains of flexibility studied here (compared to investigating brain activity during moral flexibility only and correlating this activity with multiple types of flexible behaviour outside the scanner). Nevertheless, as we hypothesized, the role of TPJ in attention-shifting might be the most plausible candidate to explain our findings (mediation effects of TPJ observed in the three flexible domains). In fact, it would be difficult to interpret our findings by other domain-specific cognitive functions, that is, the association between TPJ activity during moral flexibility and the level of perceptual or economic flexibility outside the scanner.

In this view, our interpretation of such functional causality is highly speculative, as there are several confounding factors. For example, individuals’ level of behavioural flexibility could be influenced by their level of cognitive/emotional intelligence, social/empathic skill, age, and gender^47–49^. They may have various impacts on different domains of flexibility. Thus, caution should be taken concerning the overgeneralization of behavioural flexibility. Therefore, given such a preliminary nature of the study, we suggest that a future study should take these confounding factors more strictly into account. In addition, more studies are certainly required for robustly replicating the possible link between the individuals’ skill of different flexibility and strength of brain activity, for which we found modest levels of these relationships.

Our findings suggest that focusing on lower-level attention-shifting might provide new avenues towards gaining a better understanding of behavioural flexibility, given that optimal levels of arousal and social motivation are conducive to flexible behaviour^50^. Behavioural flexibility might rely on more basic attention-shifting that helps the adoption of different domains of ‘perspectives’.

## Methods

### Subjects

Twenty-four students participated in the study (7 females, mean age = 21.3 ± 1.2 years). Exclusion criteria included a history of neurological injury or disease, serious medical/surgical illness, and substance abuse. The subjects did not meet criteria for psychiatric disorders as measured by the Structured Clinical Interview for DSM-IV Axis I Disorders (SCID I). All subjects provided informed written consent and were compensated for their participation. This study was approved by the Committee on Medical Ethics of Kyoto University and carried out in accordance with The Code of Ethics of the World Medical Association.

### Moral Dilemma task (MD)

The subjects first underwent an MRI scan during the MD task. MD is a well-established measure that manipulates conflicts between concerns for personal welfare and concerns for others’ welfare, thereby enabling the observation of moral flexibility^51^ via attention-shifting^17^. MD was a block design task that included a set of 40 moral and 20 non-moral (control) scenarios. Moral scenarios were designed so that the subjects would feel that the scenarios were morally wrong, but that it was potentially acceptable to conduct the action in real life. MD included 15 blocks (24 sec each). Each block included four trials (6 sec each), where subjects viewed short phrases representing each scenario. The subjects read each scenario silently, imagining that they were the protagonist. For moral scenarios, the subjects were instructed to press a button to either evaluate: 1) whether the action was morally right or wrong (R/W condition; 5 blocks), or 2) whether or not they would actually conduct the action in real life, considering the costs and benefits of each action (C/B condition; 5 blocks), in addition to R/W. For non-moral scenarios, they were instructed to press a button to evaluate whether or not (yes/no) they agreed with each statement (Y/N condition; 5 blocks). Further, a fixation cross was displayed between respective blocks for 14 sec. Therefore, in this task, the subjects were always evaluated by the same scenarios twice (R/W and C/B). To avoid a confound effect, these R/W, C/B, and Y/N conditions were displayed in a pseudo-random order, that is, exactly the same order of questions for every subject. Specifically, while three blocks of moral scenarios were displayed with R/W evaluation first (before C/B), two blocks of moral scenarios were displayed with C/B evaluation first (before R/W). To investigate brain patterns during flexible decision-making, we examined the brain regions activated more strongly in C/B compared to R/W (i.e., conflict resolution contrast). After MRI scanning, a post-scan rating interview was performed; the subjects were shown the same stimuli (i.e., 60 stimuli in total) and instructed to use a 7-point Likert scale to rate the level of conflict they felt (1 = ‘felt no sense of conflict at all’, 7 = ‘felt extreme sense of conflict’). Sense-of-conflict scores were used in the other analyses that will be reported elsewhere.

### Ultimatum Game (UG)

The subjects completed UG and WCST outside the MRI scanner. UG is a well-established measure that manipulates conflicts between financial interests and fairness/justice interests, thereby enabling the observation of economic flexibility^52^. The subjects played the role of responder in a two-person UG against an anonymous, computerized proposer (age- and gender-matched imaginary subject). In this computer task, the proposer offered to split a sum of ten coins (i.e., 100 Japanese yen with one coin corresponding to 10 Japanese yen, or approximately 0.10 US dollars) with the subject (i.e., responder). The subject was told that if he/she accepted the offer, both the proposer and the responder would be paid accordingly, but if the subject rejected the offer, neither the proposer nor the responder would receive any payment. Subsequently, we calculated the subjects’ acceptance rates (AR) for fair and unfair offers. In the current study, we only examined AR of unfair offers. In the manner of previous studies, each trial was performed with a new proposer to avoid learning and reputation effects^53^, and only the first names of the responders (subjects) and the proposers were displayed on the computer screen to ensure anonymity. In addition, before the experimental session, participants practiced the task until they understood it thoroughly, and they were also given the explanation that the offers of the proposers had been obtained in an earlier part of the study by other real subjects who participated in another experiment^33^. The subjects were also informed that at the end of the task, the computer would randomly select three trials and compute their earnings, and these payments would be added to their final compensation. In reality, all subjects received the maximum earnings.

### Wisconsin Card Sorting Test (WCST)

WCST is a well-established measure for cognitive/perceptual flexibility, which assesses the ability to shift attentional sets across various perceptual categories. The current study adopted a computerized version of WCST^54^. In this test, we focused on the number of categories achieved (CA), which represented a perceptual domain of flexibility. One CA represented one rule attainment counted by 6 consecutive correct selections after the rule changes. Therefore, larger CAs represent greater flexible decision-making within the perceptual domain^5^. Note that WCST data of one subject was omitted because of a technical error.

### fMRI data acquisition, preprocessing & analysis

All subjects completed an MRI scan with a 3-T scanner (Verio, Siemens, Erlangen, Germany) equipped with a 32-channel phased-array head coil. Functional images were obtained using a T2*-weighted gradient echo-planar imaging (EPI) sequence with the following parameters: TE/TR: 25/2000 ms, flip angle = 75°, field of view (FOV) = 224 × 224 mm, 34 interleaved axial slices without gaps, resolution = 3.5 mm cubic voxels. To allow for signal stabilization, the first two volumes were not saved, but the subsequent 292 volumes were then acquired. To reduce image distortions and signal loss caused by susceptibility gradients in the orbitofrontal cortex, we used a tilted acquisition to the AC-PC line. This method optimized the imaging slice orientation^55^. Each subject lay supine on a scanner bed, with a button-response device held in the right hand. MRI-compatible glasses were used to correct the vision of subjects with poor eyesight, and foam padding was used to reduce head motion. Visual stimuli were back-projected onto a screen through a built-in mirror in the scanner bore. Imaging data were preprocessed and analyzed using SPM 8 (Statistical Parametric Mapping: Wellcome Department of Imaging Neuroscience, London, UK). Functional images were corrected for differences in slice-acquisition timing and then spatially realigned to correct for head motion. Realigned images were spatially normalized to fit the EPI template supplied by SPM 8. These images were resampled into 2 mm × 2 mm × 2 mm voxels during the normalization process. All EPI images were smoothed using a Gaussian kernel with full-width at half-maximum of 8 mm in the x, y, and z-axes to increase the signal-to-noise ratio. Time-series data were high-pass filtered at 128 s to remove low frequency drifts.

### General linear model

After pre-processing, a separate general linear model was constructed for each subject. We fitted a general linear model (GLM)^56^ to the fMRI data. In the first-level analyses, the design matrix included the R/W, C/B, and Y/N conditions, in addition to the task-instruction periods. We also included the six movement parameters (three displacements and three rotations) to minimize motion-related artifacts as regressors of no interest. The conflict resolution contrast was identified as the difference between the C/B and R/W conditions (C/B > R/W). These single subject contrasts were used for the second-level fMRI analyses. Specifically, in this second-level analyses, main effects of conflict resolution were calculated by one-sample t-tests using a random-effects model.

### Whole-brain group analysis

We conducted whole-volume voxel-wise analysis. The statistical threshold was defined at cluster-level of *p* < 0.01 with a family-wise error (*FWE*) correction for multiple comparisons (at voxel-level uncorrected p < 0.001). We interpreted the anatomical location of the clusters by consulting neuroanatomy atlas books, MRIcron (http://people.cas.sc.edu/rorden/mricron/index.html), and SPM8 extension XjView (http://www.alivelearn.net/xjview).

### Region of interest analysis

Based on our literature-based hypothesis, we then performed a priori region of interest (ROI) analyses to further determine whether neural activity within both of our predefined relevant brain regions (i.e., R-DLPFC and R-TPJ) respond with regard to conflict-resolution effect (C/B > R/W). R-TPJ and R-DLPFC ROIs were limited to the right hemisphere to study intra-hemispheric relationships. This was because the right hemisphere has been the one most frequently involved in the ventral attention orienting network^13, 23, 39^ as well as in social cognition^16, 57^. Based on a previous study^58^, using the Wake Forest University (WFU) PickAtlas toolbox^59^, we applied a mask including the right BA 46 for R-DLPFC ROI, and a standard 10-mm sphere mask^60, 61^ for R-TPJ ROI [x-y-z Talairach coordinates (+50, − 55, 25)] obtained in the meta-analysis for mentalizing system involving perspective shifting towards other individuals via attention orientation^62, 63^. This procedure was based on ROI-reporting policies^64^, suggesting that while it is possible to take the stereotactic coordinates from an activation in a single/localizer study and place an ROI at that location, it is also recommendable to derive a ROI from meta-analyses. We defined activity as significant if it survived family-wise error (*FWE*) correction for multiple comparisons, with a cluster-level of *p* < 0.01 (at voxel-level uncorrected p < 0.001), in accordance with previous ROI studies^65^. The parameter estimates were extracted as first eigenvariates from the significant clusters within these ROIs, and these were used for the following analyses consistently. The VOI function in SPM8 was used to extract the parameter estimates from the significant clusters.

### Mediation analyses

The mediation effect was tested using SPSS macro ‘Indirect^66^’, which examines whether an independent variable impacts a dependent variable through mediators. We tested the significance of the mediation effect by bootstrapping strategy within this macro. This bootstrapping method repeatedly extracts samples from the dataset and estimates the mediation effect in each resampled dataset. Rather than providing conventional p values, this resampling method constructs an approximation of the sampling distribution and constructs confidence intervals. Using this model, if a confidence interval does not contain zero, then the mediated effect is considered significant^66^. While the minimum recommended number of samples to extract using this bootstrapping is 1000, we applied 5000 samples to obtain more robust confidence intervals. Moreover, we applied this statistical test with the strictest threshold using ‘bias-corrected/accelerated confidence intervals’. Furthermore, we calculated a composite score of the behavioural flexibility measures of three domains (moral, economic, and perceptual) by averaging the normalized scores. Subsequently, we conducted mediation analysis using this composite score.

### PPI analysis

As an additional analysis, we examined functional connectivity between R-TPJ and R-DLPFC using psychophysiological interaction analysis^67^. Details are described in the Supplementary Methods.

For more detail of Methods, see Supplementary information.

## Electronic supplementary material

Supplementary Information
