# Supplementary material for: Collaborative roles of Temporoparietal Junction and Dorsolateral Prefrontal Cortex in Different Types of Behavioural Flexibility
Source: Sci Rep. 2017 Jul 25;7:6415. doi: 10.1038/s41598-017-06662-6 (PMC5526981; doi:10.1038/s41598-017-06662-6)
Supplement: Supplementary file 1 — Supplementary Information [file 41598_2017_6662_MOESM1_ESM.pdf]

Supplementary Information

Collaborative roles of Temporoparietal Junction and Dorsolateral Prefrontal Cortex in Different Types of Behavioural Flexibility

Authors:

Shisei Tei[1,2,3†], Junya Fujino[1,4†], Ryosaku Kawada[1†], Kathryn F. Jankowski[5], Jukka-Pekka Kauppi[6,7], Wouter van den Bos[8], Nobuhito Abe[9], Genichi Sugihara[1], Jun Miyata[1], Toshiya Murai[1], Hidehiko Takahashi[1]*

Affiliations:

1. Department of Psychiatry, Kyoto University Graduate School of Medicine, Sakyo-ku, Kyoto 606-8507, Japan
2. Institute of Applied Brain Sciences, Waseda University, 2-579-15 Mikajima, Tokorozawa, Saitama 359-1192, Japan
3. School of Human and Social Sciences, Tokyo International University, Saitama, 350-1198, Japan
4. Medical Institute of Developmental Disabilities Research, Showa University Karasuyama Hospital, Tokyo 157-8577, Japan
5. Department of Psychology, University of Oregon, Eugene, OR, 97403, USA
6. Department of Mathematical Information Technology, University of Jyväskylä, Jyväskylä, Finland
7. Department of Computer Science and HIIT, University of Helsinki, P.O. 68 (Gustaf Hällströmin katu 2b), FI-00014, Helsinki, Finland
8. Center for Adaptive Rationality, Max Planck Institute for Human Development, 14197 Berlin, Germany
9. Kokoro Research Center, Kyoto University, Sakyo-ku, Kyoto 606-8507, Japan

†These authors have contributed equally to this work.

***Corresponding author:** Hidehiko Takahashi
Department of Psychiatry, Kyoto University Graduate School of Medicine, Sakyo-ku, Kyoto 606-8507, Japan
Tel: +81-75-751-3386, Fax: +81-75-751-3246
E-mail: hidehiko@kuhp.kyoto-u.ac.jp

Supplementary Methods

Subjects

Among the twenty-four students participating in this study, twenty-two were right-handed and two were ambidextrous. Exclusion criteria included a history of neurological injury or disease, serious medical/surgical illness, and substance abuse. Further, all subjects underwent structural MRI scanning to rule out significant cerebral anatomic abnormalities. This study was approved by the Committee on Medical Ethics of Kyoto University (permission number: E1924) and carried out in accordance with The Code of Ethics of the World Medical Association. In this study, we adopted one-tailed significance tests, which reflect our a priori unidirectional hypotheses.

Ultimatum Game (UG)

UG task was designed based on a previous study consisting of 20 trials per subject[1]. Following the conventional implementation of this study[1], we also established five types of offers. That is, the proposer offered the responder 50, 40, 30, 20 or 10 yen. Moreover, we kept the same offering ratio with this study: offers of 50 and 40 yen (50% and 40% of 100 yen) were regarded as fair offers, and those of 30, 20, and 10 yen (30%, 20%, and 10% of 100 yen) were regarded as unfair offers. Accordingly, fair offers (40% of total offers: 8 offers) and unfair offers (60% of total offers: 12 offers) were presented randomly.

Wisconsin Card Sorting Test (WCST)

During WCST, four stimulus cards were displayed on the computer screen. These cards varied according to three categories: the number, colour, and shape of their elements. The subjects were instructed to select one of the four cards, but were not told which perceptual category to use in order to choose the card. After each selection, there was a feedback (‘Right’ or ‘Wrong’) on the screen. The ‘Right’ category shifted among the three categories (number, colour, and shape) during the test, which continued until the subjects had selected all 48 cards.

Psychophysiological interaction analysis

To investigate whether R-TPJ and R-DLPFC interact to adjust attention allocation during flexible decision-making, we conducted Psychophysiological interaction (PPI) analysis. We used the generalized PPI (gPPI) toolbox, which has increased sensitivity and flexibility of statistical modeling (http://www.nitrc.org/projects/gppi/). In gPPI, deconvolved activity of the seed region is assigned to separate regressors dependent on the psychological conditions and reconvolved with the hemodynamic response function. Following a conventional study, deconvolved time series of R-TPJ activity (x, y, z = 48, -52, 18) were obtained for each subject as a seed region[2]. Based on the manual, we included 1) C/B; 2) R/W; and 3) Y/N conditions in the analysis. Contrast images associated with the PPI regressor were then entered into a regression analysis. The PPI contrast compared the C/B condition*R-TPJ (contrast weight: +1) with the R/W condition*R-TPJ (contrast weight: -1). Based on previous studies[3,4], the statistical threshold was set at corrected cluster-level $p < 0.01$ (individual voxel $p < 0.05$, cluster size ≥ 193 voxels) to reduce the risk of false negatives[5]. This threshold was obtained by

Monte Carlo simulations using Gaussian random field theory multiple comparison correction that applied 10,000 iterations within the R-DLPFC [REST (http://restfmri. net/forum/rest)]. Specifically, based on the manual, Gaussian filter widths were determined as 7.681, 8.757, 11.481 (FWHMx, FWHMy, FWHMz, in mm), and the cluster connectivity criterion was set at 3 (rmm). The risk of getting an inappropriate clustering threshold may increase by the amount of smoothing of the observed fMRI signals (i.e., degree of false positives). Namely, immoderate smoothing enhances spatial correlation of the fMRI noise across voxels, which is characterized by a spatial autocorrelation function that assumes a Gaussian shape across the whole brain. In effect, this would result in a slight overestimation of the clustering threshold[6,7], in which the degree of overestimation may vary marginally depending on each dataset[8].

In this analysis, we expected either positive or negative connectivity between TPJ and DLPFC in the conflict resolution contrast (C/B > R/W), which represented greater flexibility. This is because both positive and negative connectivity between two brain regions may potentially be accompanied by co-activation in GLM group-level activation analyses (i.e., increased hemodynamic activity). In other words, positive connectivity between two regions (accompanied by co-activation) can be due to these regions working in concert[9]. Meanwhile, negative connectivity (accompanied by co-activation) can be due to reciprocal modulation[9]. More specifically, in an activation study, a prediction of activity in one brain region by greater activity in another region indicates functional integration, whereas negative connectivity between these regions may further suggest a dynamic interplay via reciprocal modulation[10]. Accordingly, the results of the

PPI analyses represent the strength of regression of activity in one region on another (i.e., the level of activity in two regions correlates over time). That is, activity in one region may be driven/explained by activity in the other, and that hemodynamic activity in these two regions can increase and decrease in synchronization/coupling[9]. In this respect, either positive or negative connectivity of R-TPJ and R-DLPFC, accompanied by co-activation in activation analyses, may represent behavioural flexibility via collaboration or reciprocal modulation of these brain regions.

Supplementary Results and Discussion

The results of PPI analysis showed a significant decrease in TPJ-DLPFC functional connectivity in the C/B compared to the R/W condition in the conflict resolution contrast (C/B > R/W; $p < 0.01$, cluster-level corrected; Supplementary Table S3). This negative functional connectivity was in agreement with findings from the GLM group-level activation analysis, which showed greater simultaneous TPJ and DLPFC activity (i.e., co-activation) in this contrast. These results suggest a functional integration between these regions. Specifically, our findings of negative connectivity, accompanied by co-activation of these regions (observed in the activation analysis) might imply that TPJ and DLPFC modulate each other in a reciprocal manner via bi-directional inhibitory processing[11]. In effect, we speculate that this might facilitate thinking about conflicting perspectives simultaneously, which involves both maintaining and shifting between decision-rules[12-14]. However, to consider this issue, the threshold in the PPI analysis is relatively liberal for arguing robustly about the interaction of the link between TPJ and DLPFC. Furthermore, statistical procedure of this PPI at the group-level is incompatible with that of the activation analysis. To argue about the role of the TPJ-DLPFC link in flexible behaviour, additional experiments that are especially focused on the interaction between TPJ and DLPFC should be conducted in future studies.

Supplementary Tables

Supplementary Table S1. fMRI results of whole-brain analysis

MNI (mm)			*t*		Brain Regions	Clusters k	
x	y	z				#	(voxels)
12	-92	-2	13.52	R	primary visual cortex	1	50679
-16	-92	2	11.95	L	secondary visual cortex		
-20	-60	22	11.01	L	posterior cingulate cortex		
38	-82	-10	10.21	R	associative visual cortex		
-22	-34	12	9.55	L	hippocampus		
-4	-76	-24	8.48	L	cerebellum		
-42	-54	-24	8.48	L	fusiform gyrus		
16	-60	26	8.15	R	posterior cingulate cortex		
-14	24	4	7.65	L	caudate		
2	-60	4	7.63	R	cerebellum		
-4	-78	40	7.55	L	precuneus		
42	-56	-30	7.27	R	fusiform gyrus		
52	-44	20	6.93	R	temporoparietal junction		
32	-32	8	6.79	R	extra nuclear		
54	2	-24	6.44	R	temporopolar area		
-56	-46	4	6.17	L	middle temporal gyrus		
2	42	-16	5.99	R	orbito frontal cortex		
-28	26	-22	5.85	L	orbito frontal cortex		
-30	-62	0	5.80	L	associative visual cortex		
36	-22	-16	5.39	R	hippocampus		
4	2	66	6.72	R	supplementary motor cortex	2	4507
-50	8	50	5.96	L	supplementary motor cortex		
-52	30	26	5.74	L	dorsolateral prefrontal cortex		
-32	-8	42	4.50	L	precentral gyrus		
2	28	52	4.27	R	medial frontal cortex		
16	50	48	5.80	R	dorsolateral prefrontal cortex	3	907
32	58	28	4.07	R	frontal polar area		
30	12	28	5.60	R	dorsolateral prefrontal cortex	4	1101
-26	60	8	5.22	L	frontal polar area	5	409

Regions showing activity in conflict resolution contrast. Results are reported at threshold of cluster-level of $p < 0.01$ with family-wise error (*FWE*) correction for multiple comparisons (at voxel-level uncorrected $p < 0.001$). Abbreviations: BA=Brodmann Area, FWE = family-wise error, L = left, MNI = Montreal Neurological Institute, R = right

Supplementary Table S2. Brain activity within R-TPJ and R-DLPFC ROIs during C/B > R/W contrast of moral dilemma task (MD).

**ROIs**	MNI	t	Z	k
R-DLPFC	30, 12, 28	5.60	4.41	329*
R-TPJ	48, -52, 18	5.01	4.08	101*

*p < 0.01 (cluster-level, *FWE*-corrected).

Supplementary Table S3. Functional connectivity between R-TPJ and R-DLPFC by PPI analysis.

	**x y z**	t	k
R-DLPFC	52, 24, 26	2.92	339

There was negative condition-dependent connectivity between R-TPJ and R-DLPFC in C/B compared to R/W condition.

Supplementary Figure

Supplementary Figure S1. Whole-brain statistical maps showing group activation.

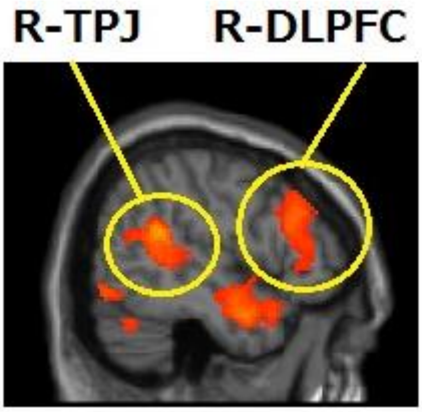

R-TPJ and R-DLPFC activity was observed at whole-brain *FWE* cluster-level corrected $p < 0.01$ (at voxel-level uncorrected $p < 0.001$).

Supplementary Information References

1. Takahashi, H., et al. Honesty mediates the relationship between serotonin and reaction to unfairness. *Proceedings of the National Academy of Sciences of the United States of America* 109, 4281-4284 (2012).
2. Sasse, L.K., Peters, J., Buchel, C. & Brassen, S. Effects of prospective thinking on intertemporal choice: The role of familiarity. *Human Brain Mapping* 36, 4210-4221 (2015).
3. Zou, Q., et al. Functional connectivity between the thalamus and visual cortex under eyes closed and eyes open conditions: a resting-state fMRI study. *Human Brain Mapping* 30, 3066-3078 (2009).
4. Wang, Z., et al. Baseline and longitudinal patterns of hippocampal connectivity in mild cognitive impairment: evidence from resting state fMRI. *Journal of the Neurological Sciences* 309, 79-85 (2011).
5. Lieberman, M.D. & Cunningham, W.A. Type I and Type II error concerns in fMRI research: re-balancing the scale. *Social Cognitive Affective Neuroscience* 4, 423-428 (2009).
6. Cox, R.W., Reynolds, R.C., and Taylor, P.A. AFNI and Clustering: False Positive Rates Redux. bioRxiv, 065862 (2016).
7. Eklund, A., Nichols, T.E. & Knutsson, H. Cluster failure: Why fMRI inferences for spatial extent have inflated false-positive rates. *Proceedings of the National Academy of Sciences of the United States of America* 113, 7900-7905 (2016).
8. Bennett, C.M., Wolford, G.L. & Miller, M.B. The principled control of false positives in neuroimaging. *Social Cognitive Affective Neuroscience* 4, 417-422 (2009).
9. O'Reilly, J.X., Woolrich, M.W., Behrens, T.E., Smith, S.M. & Johansen-Berg, H. Tools of the trade: psychophysiological interactions and functional connectivity. *Social Cognitive Affective Neuroscience* 7, 604-609 (2012).
10. Friston, K.J., et al. Psychophysiological and modulatory interactions in neuroimaging. *Neuroimage* 6, 218-229 (1997).
11. Longe, O., Senior, C., and Rippon, G. The Lateral and Ventromedial Prefrontal Cortex Work as a Dynamic Integrated System: Evidence from fMRI Connectivity Analysis. *Journal of Cognitive Neuroscience* 21, 141-154 (2008).
12. Pearson, J.M., Heilbronner, S.R., Barack, D.L., Hayden, B.Y. & Platt, M.L. Posterior cingulate cortex: adapting behavior to a changing world. *Trends in Cognitive Science* 15, 143-151 (2011).
13. Esterman, M., Noonan, S.K., Rosenberg, M. & Degutis, J. In the zone or zoning out? Tracking behavioral and neural fluctuations during sustained attention. *Cerebral Cortex* 23, 2712-2723 (2013).

14. Sali, A.W., Courtney, S.M. & Yantis, S. Spontaneous Fluctuations in the Flexible Control of Covert Attention. *Journal of Neuroscience* 36, 445-454 (2016).

15. Duvernoy, H.M. The Human Brain. Surface, Three-dimensional Sectional Anatomy and MRI, (Springer-Verlag, 1991).

16. Talairach, J., and Tournoux, P. Co-planar Stereotaxic Atlas of The Human Brain: 3-Dimensional Proportional System —An Approach to Cerebral Imaging, (Thieme, New York, 1988).
